# A proposed approach for standardized reporting of data linkage processes and results.

**DOI:** 10.23889/ijpds.v7i3.1962

**Published:** 2022-08-25

**Authors:** Yinshan Zhao, Mike Jarrett, Kimberlyn McGail, Brent Hills

**Affiliations:** 1 Population Data BC; 2 UBC School of Population and Public Health and Centre for Health Services and Policy Research

## Objectives

Population Data BC (PopData) is an agency in British Columbia, Canada, that routinely performs linkages of various administrative and researcher-collected data to a population spine. We developed a linkage report template in order to increase transparency of linkage process and outcome for end users and data providers.

## Approach

PopData performs probabilistic and deterministic data linkage using an in-house software. A literature review identified existing guidelines and examples of linkage reporting. A survey collected input from a wide range of end users about their interest in receiving linkage reports and specific information that is important to their work. A draft template was developed by PopData’s linkage experts and data scientists which then was reviewed by PopData staff and external partners. Privacy requirements, mode of delivery, readability to the intended audience and operational feasibility were carefully considered.

## Results

The resulting template built on our existing internal linkage summaries. The report follows a framework suggested in the literature with three key components: 1) information on the data source and linkage fields, 2) data pre-processing and linkage methodology, and 3) linkage results, presented in tables and figures, including overall linkage rates, detail on matched fields, and the distribution of linkage weights of linked and unliked pairs. In addition, an appendix describes the linkage methods and population spine in detail, and supplementary notes will comment on unique issues related to the data, when those are applicable. Educational materials to aid understanding of linkage methodologies and reporting are also under development.

## Conclusion

Linked data are increasingly used in research, making it important to provide information on linkage process and performance to the research community. Rigorous and standardized linkage reports produced by data centres can facilitate evaluation of the impact of linkage performance on research findings and enable transparent reporting in peer-reviewed research.

